# Gender Differences in Metabolic Disorders and Related Diseases in Spontaneously Diabetic Torii-*Lepr* ^*fa*^ Rats

**DOI:** 10.1155/2014/841957

**Published:** 2014-05-08

**Authors:** Takeshi Ohta, Yoshiaki Katsuda, Katsuhiro Miyajima, Tomohiko Sasase, Shuichi Kimura, Bin Tong, Takahisa Yamada

**Affiliations:** ^1^Japan Tobacco Inc., Central Pharmaceutical Research Institute, 1-1, Murasaki-cho, Takatsuki, Osaka 569-1125, Japan; ^2^Laboratory of Animal Genetics, Graduate School of Science and Technology, Niigata University, Nishi-ku, Niigata 950-2181, Japan

## Abstract

The Spontaneously Diabetic Torii *Lepr* ^*fa*^ (SDT fatty) rat is a novel type 2 diabetic model wherein both male and female rats develop glucose and lipid abnormalities from a young age. In this study, we investigated gender differences in abnormalities and related complications in SDT fatty rats. Food intake was higher in males compared to female rats; however, body weight was not different between genders. Progression of diabetes, including increases in blood glucose and declines in blood insulin, was observed earlier in male rats than in females, and diabetic grade was more critical in male rats. Blood lipids tended to increase in female rats. Gonadal dysfunction was observed in both male and female rats with aging. Microangiopathies, such as nephropathy, retinopathy, neuropathy, and osteoporosis, were seen in both genders, and pathological grade and progression were more significant in males. Qualitative and quantitative changes were observed for metabolic disease gender differences in SDT fatty rats. The SDT fatty rat is a useful model for researching gender differences in metabolic disorders and related diseases in diabetes with obesity.

## 1. Introduction

Recently, gender-specific medicine (GSM) has become an active field in current medical care. The field of GSM examines how normal human biology and physiology differ between males and females and how diagnosis and treatment of diseases differ as a function of gender. Males and females differ in their experience of diabetes mellitus. For optimal prevention and treatment of the disease, these differences must be acknowledged [1–3]. Some studies have shown that serum endogenous sex hormone levels are related to type 2 diabetes, insulin resistance, and other components of metabolic syndrome [4, 5].

The Spontaneously Diabetic Torii *Lepr* ^*fa*^ (SDT fatty) rat, established by introducing the* fa* allele of the Zucker fatty rat into the SDT rat genome, is a new model of obese type 2 diabetes. Both male and female SDT fatty rats showed overt obesity, and hyperglycemia and hyperlipidemia were observed at a young age as compared with SDT rats [6, 7]. Female rats have the potential to become an animal model of type 2 diabetes with obesity for women, for which few models currently exist [8]. In this study, we investigated gender differences in glucose and lipid abnormalities and related diseases in SDT fatty rats. New findings of gonadal dysfunction in male and female SDT fatty rats were reported in Section 3.

## 2. Gender Differences in Glucose and Lipid Abnormalities

### 2.1. Materials

In male and female SDT fatty rats, body weight and blood biochemistry parameters, such as glucose, insulin, triglycerides (TG), and total cholesterol (TC), were periodically examined from 5 to 20 weeks of age. Blood samples were collected from the tail vein of nonfasted rats. Serum glucose, TG, and TC levels were measured using commercial kits (Roche Diagnostics, Basel, Switzerland) and an automated analyzer (Hitachi, Tokyo, Japan). Serum insulin level was measured with a rat-insulin enzyme-linked immunosorbent assay (ELISA) kit (Morinaga Institute of Biological Science, Yokohama, Japan).

### 2.2. Gender Differences in Diabetic Models

**Figure 1:**
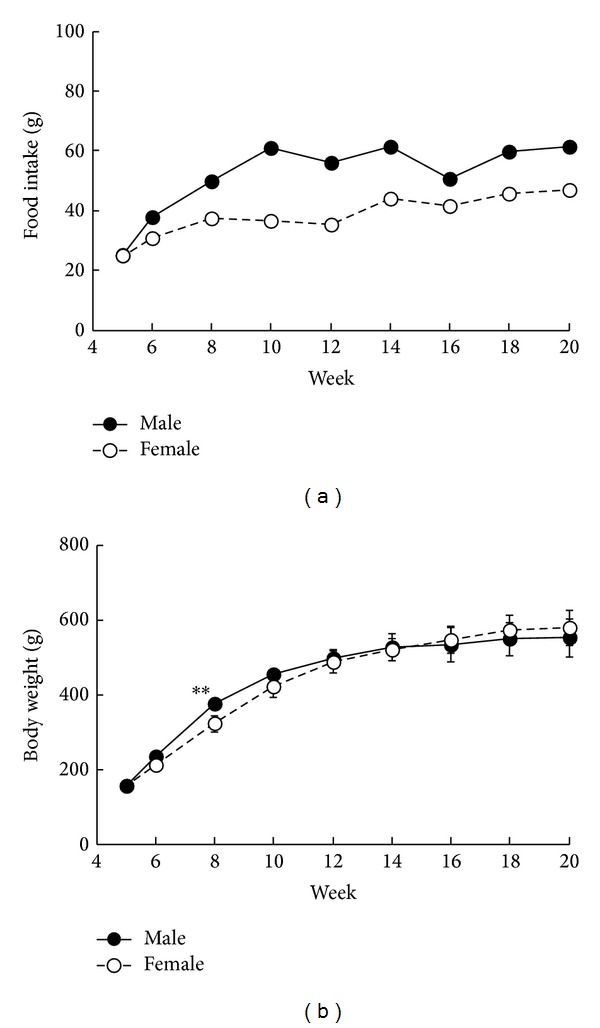
Changes in food intake (a) and body weight (b) in male and female SDT fatty rats. Data are shown as mean (a) or means ± standard deviation (b) (*n* = 5). Statistical analysis of differences between mean values was performed using the* F*-test, followed by Student's* t*-test or Aspin-Welch's* t*-test. *P < 0.05 and **P < 0.01, significantly different from the female SDT fatty rats.

**Figure 2:**
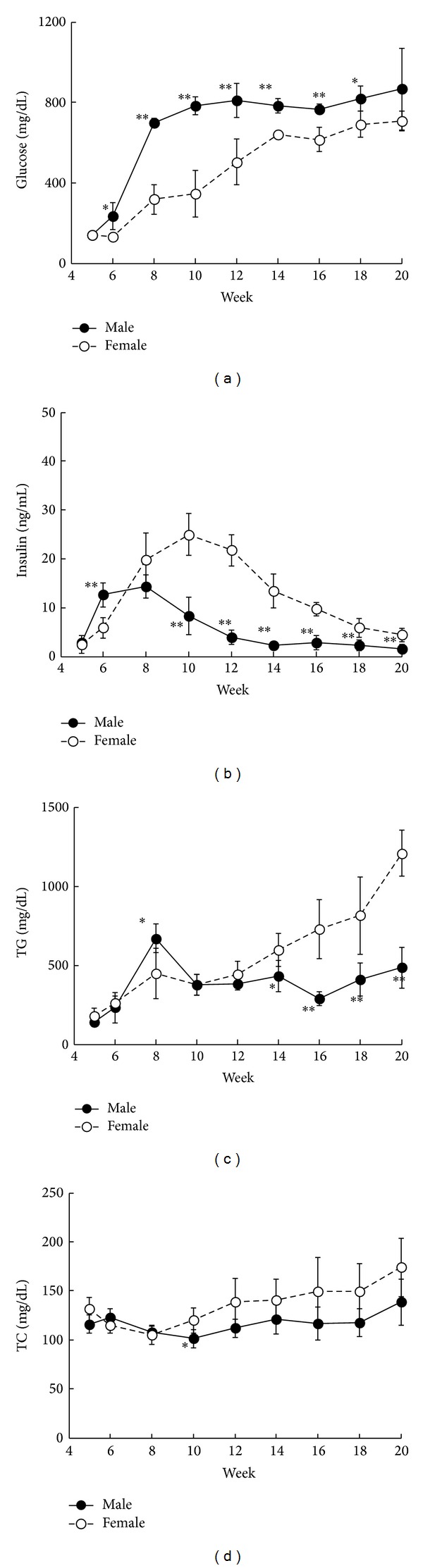
Changes in biological parameters ((a) glucose, (b) insulin, (c) triglyceride (TG), and (d) total cholesterol (TC)) in male and female SDT fatty rats. Data are shown as means ± standard deviation (*n* = 5). *P < 0.05 and **P < 0.01, significantly different from the female SDT fatty rats.

Food intake was higher in male SDT fatty rats after 6 weeks of age, as compared with intake observed in female rats (Figure 1(a)). Changes in body weight were almost comparable in both male and female rats; however, body weight in male rats only increased at 8 weeks of age (Figure 1(b)). In addition, visceral fat weight was determined in male and female rats at 16 weeks of age using computed tomography (CT) analysis (LATheta, ALOKA Co., Ltd., Osaka, Japan) (mean ± standard deviation: male rats, 69.5 ± 8.6 g and female rats, 75.1 ± 8.2 g), and results showed that levels were higher in fatty rats, regardless of gender, as compared with lean rats. Blood glucose levels in male rats were remarkably elevated after 6 weeks of age, and hyperglycemia was observed in female rats after 8 weeks of age (Figure 2(a)). Hyperglycemia observed in male rats was more significant than that in female rats. Due to the severe hyperglycemia observed in male rats, body weight in male and female rats was comparable despite differences in food intake. Blood insulin levels in male rats increased from 6 to 10 weeks of age as compared with those in female rats; however, insulin levels rapidly decreased after 10 weeks of age (Figure 2(b)). Since hyperglycemia observed in male rats was more severe than observed in female rats, the decline in insulin level was considered more pronounced compared to female rats. The severe decline in insulin levels in male rats was considered as leading to the stagnation of body weight gain despite hyperphagia (Figure 1). A gender difference was observed in the onset of diabetes in SDT rats [9], and the reason may be partly attributed to estrogen, which inhibits the development of diabetes. Female SDT fatty rats were considered as maintaining higher insulin levels, which suggests a possible pancreatic protection effect, as compared with male rats for similar reasons. Blood TG levels in male rats were temporarily elevated at 8 weeks of age as compared with those in female rats, whereas TG levels after 14 weeks of age were significantly higher in female rats (Figure 2(c)). Moreover, a tendency towards increases in blood TC levels in female rats was observed after 10 weeks of age, as compared with those in male rats (Figure 2(d)). Glucose levels were higher in males than in female rats, whereas lipid levels were higher in females than in male rats.

Gender differences in diabetes were reported in other diabetic models. Hyperglycemia was more often observed in younger male rats than in female rats in some diabetic rat models. In Zucker diabetic fatty rats, diabetes developed in male rats after 9 weeks of age, whereas hyperglycemia was not seen in female rats until 22 weeks of age [10]. Male Otsuka Long-Evans Tokushima fatty (OLETF) rats developed diabetes after 18 weeks of age and the incidence of diabetes was 100% at 25 weeks of age, whereas the incidence of diabetes in female rats was about 30% even at 60 weeks of age [11]. In both ZDF and OLETF rats, the incidence of diabetes in female rats was remarkably lower than the incidence in male rats. In Tsumura Suzuki obese diabetes (TSOD) mice, the incidence of diabetes was also 100% in male mice, whereas female mice did not develop diabetes [12]. In BALB/cA mice, males gained more body weight and body fat weight and had higher energy intake than females from high-fat diet feeding. BALB/cA female mice were resistant to HFD-induced obesity compared to males [13]. The reason for metabolic abnormalities in females being significantly smaller compared to those in males is considered due to female hormones, such as estrogen, that have a protective effect on metabolic dysfunction. It is well known that oophorectomy promotes the development of diabetes [9]. In female nonobese diabetic (NOD) mice, diabetes was observed at around 13 weeks of age and incidence gradually increased with age. In males, however, symptoms were only observed in a few animals [14]. Moreover, in Komeda diabetes-prone (KDP) rats, there were no gender differences in onset of diabetes [15].

## 3. Gender Differences in Gonadal Dysfunction

### 3.1. Materials

In male SDT fatty rats, serum testosterone level was measured with a Testosterone EIA kit (Cayman Chemical Company, MI, USA) from 8 to 40 weeks of age. Semen analyses and pathological analyses of testes were performed at 32 and 40 weeks of age. Testes with epididymis were removed, and the cauda epididymis was separated from the testis to collect semen. Squeezed semen was incubated in buffer containing BSA at 37°C for 30 minutes. Ten *μ*L of sperm suspension was dropped on a glass side, and 700–1000 sperm cells were observed for motility under a microscope using 100–400x magnification. The percentage of motile sperm cells (sperm motility) was calculated as the number of motile sperm cells divided by the total number of sperm cells. Sperm viability was evaluated with SYBR-14/propidium iodide (LIVE/DEAD Sperm Viability Kit, Life Technologies, Carlsbad, CA, USA). Live sperm ratio was defined as the number of viable sperm cells divided by the total number of sperm cells. Moreover, a total of 200–300 sperm cells per animal were randomly selected under a microscope using 400x magnification and were assessed to determine the presence or absence of morphological abnormalities. The ratio of normal sperm cells was expressed as the percentage of total sperm cell count. Necropsy was performed at 32 and 40 weeks of age. After weighing the testes and the epididymis, organs were fixed in 10% neutral buffered formalin. After resection, the tissue was paraffin-embedded by standard techniques and thin-sectioned (3 to 5 *μ*m). The sections were stained with hematoxylin and eosin (HE).

### 3.2. Gender Differences in SDT Fatty Rats

**Figure 3:**
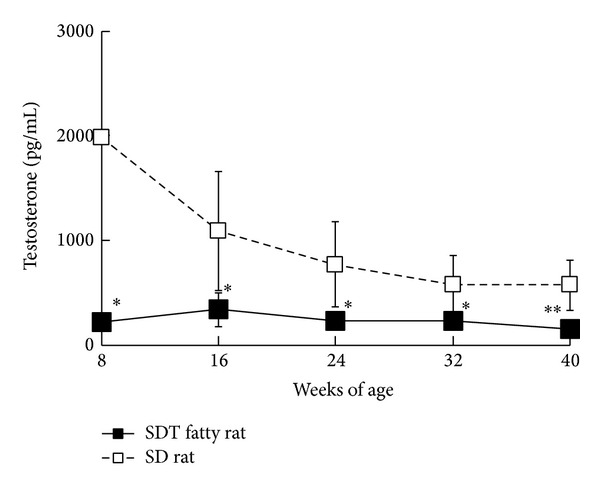
Changes in blood testosterone levels in male SDT fatty rats and SD rats. Data are shown as means ± standard deviation (*n* = 4–6). *P < 0.05 and **P < 0.01, significantly different from the SD rats.

Testosterone levels in male SDT fatty rats were decreased as compared with those in SD rats during the experimental period (Figure 3). Streptozotocin- (STZ-) induced diabetic rats showed a significant decrease in serum testosterone levels [16]; however, serum testosterone levels in Otsuka Long-Evans Tokushima fatty (OLETF) rats were comparable to those in control rats [17]. In obese males, total testosterone, free testosterone, and sex hormone-binding globulin (SHBG) levels were all commonly decreased [18, 19]. Both serum testosterone and SHBG levels were significantly lower in males with type 2 diabetes, and increasing insulin resistance was associated with decreased testosterone secretion at the testicular level (Leydig cell) [20, 21]. The decrease in serum testosterone levels in SDT fatty rats is therefore considered to be caused by several metabolic disorders, such as obesity, hyperglycemia, and insulin resistance (Figures 1 and 2). The testosterone level in SDT fatty rats was significantly lower at the start of the experimental period, when rats were 8 weeks of age. The hypotestosteronemia may be related to the prominent increase in blood glucose levels from 6 to 8 weeks of age (Figure 2(a)).

Sperm characteristics in SDT fatty rats are shown in Table 1. Sperm motility in SDT fatty rats decreased significantly at 32 weeks of age, as compared with that in SD rats. Sperm motility in SD rats decreased with aging, and no significant differences were observed between SDT fatty and SD rats at 40 weeks of age. Sperm viability in SDT fatty rats also tended to decrease at 32 weeks of age, as compared with that in SD rats; however, there were no significant differences in sperm viability at 40 weeks of age between rats. In sperm morphological analyses, the percentage of normal spermatozoa in SDT fatty rats was significantly decreased at 40 weeks of age, as compared with that in SD rats. In OLETF rats, the sperm count decreased significantly at 64 weeks of age, in comparison to control rats [17]. In humans, total sperm count and total motile sperm are negatively correlated with weight, waist circumference, and hip circumference, suggesting a potential link between obesity, hypogonadism, and infertility as indicated by semen analyses [22].

Testis and epididymis weights in SDT fatty rats are shown in Table 2. Testis and epididymis weights were determined in different experiments. The absolute weights in SDT fatty rats tended to decrease, as compared with those in SD rats, and the epididymis weight in SDT fatty rats decreased significantly at 40 weeks of age. On the other hand, a significant increase in the relative weights of testes and epididymis in SDT fatty rats compared to those in SD rats was seen, since the body weights of SDT fatty rats were lower at 32 and 40 weeks of age. In histological analyses of the testes and epididymis, no significant abnormalities were observed in SDT fatty rats at 32 and 40 weeks of age (data not shown). In the testes of STZ-induced diabetic mice, ultrastructural changes, such as an increase in lipid droplets and a decrease in smooth endoplasmic reticulum, were observed in Leydig cells [23]. A tendency toward seminiferous tubular atrophy has been reported in 64-week-old OLETF rats; however, structures for interstitial tissues including Leydig cells remained conserved [17]. No histological abnormalities were observed in testes or epididymis in SDT fatty rats, and the reason for hypotestosteronemia or sperm abnormalities should be elucidated in further studies.

**Figure 4:**
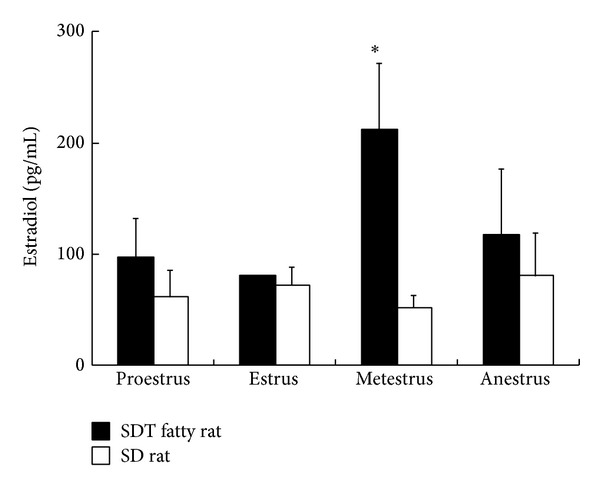
Changes in blood estradiol levels in female SDT fatty rats and SD rats at 12 weeks of age. Estradiol levels were measured in each estrus stage (proestrus, estrus, metestrus, and anestrus). Data are shown as means ± standard deviation (*n* = 4). *P < 0.05, significantly different from the SD rats.

In female SDT fatty rats, gonadal abnormalities in the estrus cycle and histopathology were reported by Inaba et al. [24]. The female rats showed an irregular estrus cycle with a persistent estrus stage, an extension of metestrus, and a large number of leukocytes in smears from the metestrus stage. Decreases in relative weights of the ovary, uterus, and vagina and histopathological changes, such as atrophy of the uterus and inflammation of the vagina, were observed in female rats at 12 weeks of age. Furthermore, blood estradiol levels in the estrus cycle were determined using an Estradiol EIA kit (Cayman Chemical Company, MI, USA) at 12 weeks of age. Estradiol levels in metestrus were increased as compared with that in SD rats, and levels tended to increase in other estrus stages (Figure 4).

In both male and female rats, gonadal dysfunction was observed with progression of metabolic disorders, such as hyperglycemia, hyperlipidemia, and hyperinsulinemia. In blood biochemistry analyses, male rats showed hypotestosteronemia, whereas female rats showed hyperestrogenemia.

## 4. Gender Differences in Microangiopathy and Osteoporosis

In both male and female SDT fatty rats, increases in renal parameters such as urine volume and urine protein were observed after 4 weeks of age [7, 8, 25]. In the renal tubules, glycogen deposition in the tubular epithelium (Armanni-Ebstein lesions) and tubular dilation were noted from 8 weeks of age in male rats, and those changes were observed from 16 weeks of age in female rats [7]. In the glomeruli, glomerulosclerosis was observed from 16 weeks of age in male rats and from 32 weeks of ages in female rats [8]. Moreover, progression of tubular and interstitial lesions, including fibrosis and inflammatory cell filtration, was observed in both male and female rats at 60 weeks of age. Nodular lesions in the glomeruli were only observed in male rats after 40 weeks of age [7].

In male SDT fatty rats after 16 weeks of age, a prolongation of the peak latencies of oscillatory potentials was observed [7] and prolongation at 22 weeks of age was observed in female rats (peak latencies of oscillatory potentials (∑(OP_1_ − OP_3_)) on electroretinogram, SDT fatty rats: 89.5 ± 3.4 ms versus SD rats: 82.5 ± 3.3 ms, *n* = 5). Histopathological findings in the lens, including hyperplasia of the epithelium, vacuolation of fibers, and formation of Morgagnian globules, were observed from 8 weeks of age in male rats, and similar changes were also observed in female rats from 16 weeks of age [7, 8]. Furthermore, retinal lesions, such as folding and thickening, were observed with aging in both male and female rats [25, 26].

Caudal motor nerve conduction velocity in both male and female SDT fatty rats was delayed at 24 weeks of age (female SDT fatty rats: 54.4 ± 8.4 m/s versus female SD rats: 66.3 ± 7.5 m/s, *n* = 6). Histopathologically, at 40 weeks of age, fiber number in male rats significantly decreased, and rats revealed significant atrophy in myelinated nerves [27]. There have been no reports in histological findings in female rats.

**Figure 5:**
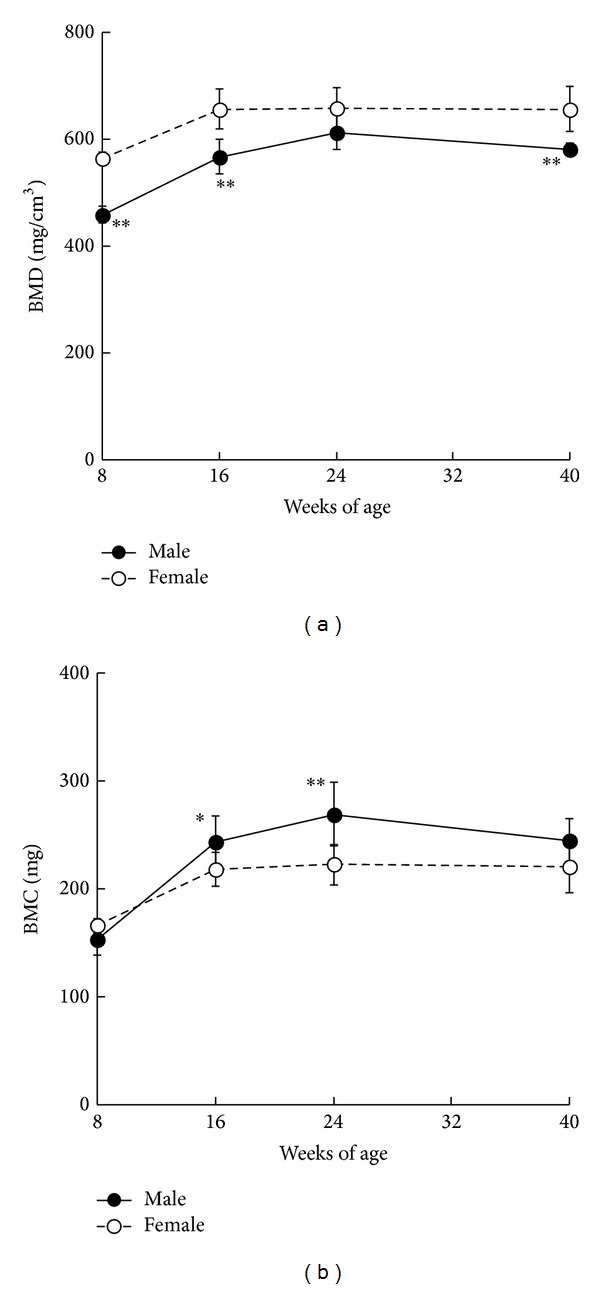
Changes in BMD (a) and BMC (b) in male and female SDT fatty rats. Data are shown as means ± standard deviation (male: *n* = 5 and female: *n* = 10). *P < 0.05 and **P < 0.01, significantly different from the female SDT fatty rats.

The effects of obese type 2 diabetes on bone metabolism were investigated in SDT fatty rats [28–30]. Both serum osteocalcin and urine deoxypyridinoline levels were lower in male rats as compared to normal rats from 8 to 40 weeks of age; however, urine deoxypyridinoline levels in female rats increased after 24 weeks of age as compared to normal rats [26]. Bone mineral density (BMD) of the whole tibia in male rats decreased as compared with that in female rats, whereas bone mineral content (BMC) in male rats increased as compared with that in female rats (Figure 5). The differences in BMD in both genders may be related to the degree of glucose and lipid metabolic disorders, and the differences in BMC may be associated with body weight.

SDT fatty rats of both genders showed metabolic abnormalities, such as hyperphagia, obesity, and increases in blood glucose and lipids, after weaning. Since hyperglycemia and hyperlipidemia were sustained for a long period afterwards, quantitatively equal changes were observed in diabetic complications in both male and female rats. On the other hand, there are gender differences in the development of diabetes in other obese diabetic models, such as ZDF rats, OLETF rats, and Wistar fatty rats, and critical findings in diabetic complications for these diabetic rats were mainly observed in male rats [25].

## 5. Conclusion

In both male and female SDT fatty rats, glucose and lipid abnormalities and related diseases were observed; however, the week of onset and severity were varied. Gender differences in pathophysiological changes seem to be based on gonadal hormones, such as testosterone and estrogen. Furthermore, pathophysiological changes in males may be associated with severe hyperglycemia, and changes in females may be related to insulin resistance as well as hyperglycemia. Gender differences in metabolic diseases in SDT fatty rats were accompanied with qualitative changes as well as quantitative changes. The SDT fatty rat is a useful model for researching the gender differences in metabolic abnormalities in diabetes with obesity.

## Figures and Tables

**Table 1:** Semen analysis in male SD and SDT fatty rats.

	32 weeks of age	40 weeks of age
Sperm motility (%)		
SD rat	79.9 ± 10.9	63.6 ± 14.7
SDT fatty rat	70.0 ± 8.2**	67.1 ± 5.0
Sperm viability (%)		
SD rat	70.9 ± 11.4	61.4 ± 12.0
SDT fatty rat	63.8 ± 5.9	67.4 ± 6.7
Sperm morphology (%)		
SD rat	84.5 ± 6.6	86.5 ± 5.3
SDT fatty rat	79.8 ± 2.5	75.3 ± 2.2**

Data represent the mean ± standard deviation (*n* = 4–6). **P < 0.01 versus age-matched SD rats.

**Table 2:** Organ weights in male SD and SDT fatty rats.

	32 weeks of age	40 weeks of age
Testes		
Absolute weight (mg)		
SD rat	4174.9 ± 341.9	3885.3 ± 102.1
SDT fatty rat	3796.6 ± 183.1	3320.3 ± 438.2
Relative weight (mg/g)		
SD rat	4.813 ± 0.758	4.072 ± 0.525
SDT fatty rat	7.578 ± 1.039**	6.294 ± 0.895**
Epididymides		
Absolute weight (mg)		
SD rat	585.8 ± 82.9	551.4 ± 43.3
SDT fatty rat	502.3 ± 6.2	494.4 ± 3.0**
Relative weight (mg/g)		
SD rat	0.837 ± 0.121	0.615 ± 0.092
SDT fatty rat	0.919 ± 0.114	0.930 ± 0.053**

Data represent the mean ± standard deviation (*n* = 4–6).

*P < 0.05 and **P < 0.01 versus age-matched SD rats.
